# Anti-Demineralization Effects of Dental Adhesive-Composites on Enamel–Root Dentin Junction

**DOI:** 10.3390/polym13193327

**Published:** 2021-09-29

**Authors:** Yu-Jung Lai, Rena Takahashi, Po-Yen Lin, Ling Kuo, Yuan Zhou, Khairul Matin, Yu-Chih Chiang, Yasushi Shimada, Junji Tagami

**Affiliations:** 1 School of Dentistry, Graduate Institute of Clinical Dentistry, National Taiwan University, Taipei 10048, Taiwan; mavislai3506@gmail.com; 2 Dental Department, Division of Restorative and Aesthetic Dentistry, National Taiwan University Hospital, Taipei 100229, Taiwan; catherine201kuo@yahoo.com; 3 Department of Cariology and Operative Dentistry, Division of Oral Health Sciences, Graduate School of Medical and Dental Sciences, Tokyo Medical and Dental University, Tokyo 113-8549, Japan; renatakahashi52@gmail.com (R.T.); zhouyuan2411@gmail.com (Y.Z.); matintmd@gmail.com (K.M.); shimada.ope@tmd.ac.jp (Y.S.); tagami.ope@tmd.ac.jp (J.T.); 4 Department of Dentistry, School of Dentistry, National Yang Ming Chiao Tung University, Taipei 112, Taiwan; matthewlin37@gmail.com; 5 Molecular Imaging Center, National Taiwan University, Taipei 10617, Taiwan

**Keywords:** adhesive composite, SPRG-filler, 10-MDP, oral biofilm reactor, swept-source-OCT, micro-CT

## Abstract

Oral biofilm reactor (OBR) and pH cycling (pHC) artificial caries model were employed to evaluate the anti-demineralization effects of four composite filling systems on enamel–root dentin junction. Sixty-four enamel–root dentin blocks (6 mm × 6 mm × 2 mm) each with a cylindrical cavity were randomly assigned to the pHC and OBR group, then four subgroups (n = 8) and filled with either the Beautifil II (BEF, SPRG-filler-containing) or Estelite (EST) composite after the adhesive (either Single Bond Universal (SBU) or FL Bond II (FL, SPRG-filler-containing)). The demineralization lesions of filling interface were examined by micro-computerized tomography (μCT) and swept-source-optical coherence tomography (SS-OCT). According to the degree of interface damage, the caries lesions were sorted into four types: Type A and B (no attachment loss); Type C and D (attachment loss). EST/SBU showed the worst demineralization lesion and attachment loss (100% Type D), while BEF/FL exhibited the shallowest lesion depth (*p* < 0.05, 145 ± 45 μm on enamel, 275 ± 35 μm on root dentin) and no attachment loss (75% Type A and 25% Type B). Using FL adhesive alone does not effectively reduce enamel demineralization. BEF plays a leading role in acid resistance. The combination of BEF and FL showed a cumulative synergistic effect on anti-demineralization.

## 1. Introduction

The applications of adhesives and resin based composite materials have rapidly gained popularity in dentistry based on the concept of minimal intervention. However, the development of recurrent caries remains one of the primary reasons for composite filling replacement [1,2]. Recurrent caries is caused by bacterial infection from either the remaining intrinsic caries or the infiltration of extrinsic cariogenic plaque. When a microleakage has occurred or the bonding interface between the composite restoration and tooth surface has been compromised, the biofilm will accumulate and enhance further bacterial growth that can quickly reach the dentinal tubules. Consequently, recurrent caries will destroy more hard tissue and further affect the pulp tissue. Recently, due to the aging of the global population, especially in developed countries, the risk of root dentin exposure to caries or recurrent caries is also increasing [3,4]. Recurrent root caries are more difficult to approach because of their location, which increases the difficulty of treatment by the dentist. Only a few commercially available dental composites claim to have anti-microbial or anti-demineralization properties. The surface pre-reacted glass-ionomer-filler (SPRG-filler) containing composite is one example of a bacteriostatic resin composite [5]. SPRG-filler is a technology that provides bioactive functions to resin composite/adhesive materials. The fluoroboroalu-minosilicate glass is the glass core of SPRG-filler, which has a unique ability to release ions such as aluminum (Al^3+^), borate (BO_3_^3−^), sodium (Na^+^), silicate (SiO_3_^2−^), strontium (Sr^2+^), and fluoride (F^−^) [6]. These ions have the capability to impart acid resistivity to enamel and promote mineralization to induce apatite formation [7,8]. Moreover, the release of multiple ions from SPRG-filler is also associated with potential antibacterial effects [5,9].

To achieve a long-lasting composite restoration, a reliable bonding interface plays a vital role in the integrity of the resin composite restored tooth structure [10]. The enzymes in saliva and the bacteria present the most challenges to the integrity of adhesive and resin composite restoration [11,12,13]. There are many methods to evaluate the interface of restorations such as dye penetration [14], confocal laser scanning microscope [15], scanning electron microscope or transmission electron microscope examination, X-ray micro-computed tomography (μCT) [16] and optical coherence tomography (OCT) or swept-source OCT (SS-OCT) [17]. Of these, only μCT and OCT or SS-OCT can assess the specimen non-destructively and three-dimensionally (3D), which enables the evaluation of the specimen from various time points. Silver nitrate dye penetration combined with micro-CT images may provide more insight to evaluate the 3D bonding interface [18], however; silver nitrate sometimes causes artifact images via X-ray irradiation. To evaluate the effect of anti-demineralization, many studies have attempted to simulate the caries process through pH cycling (pHC) or oral biofilm reactor (OBR) to create an artificial caries [19].

In this study, we aimed to investigate the anti-demineralization effects of resin composite and adhesive on adjacent enamel and root dentin. Two artificial caries models (i.e., the pHC and OBR models) were established to evaluate the interface of the resin composite restored tooth cavity (including both enamel and root dentin portion). The interface of the composite and adhesive on the adjacent enamel and root dentin were examined by μCT and SS-OCT.

We hypothesized that (1) SPRG-filler-containing composite combined with the SPRG-filler-containing adhesive will have a synergy effect of anti-demineralization on the surrounding enamel and root dentin in comparison to the non-SPRG-filler-containing the composite/adhesive restoration; and (2) the pHC model will display similar artificial caries pattern as the OBR model for both enamel and root dentin substrate.

## 2. Materials and Methods

### 2.1. Specimen Preparation

**Figure 1:**
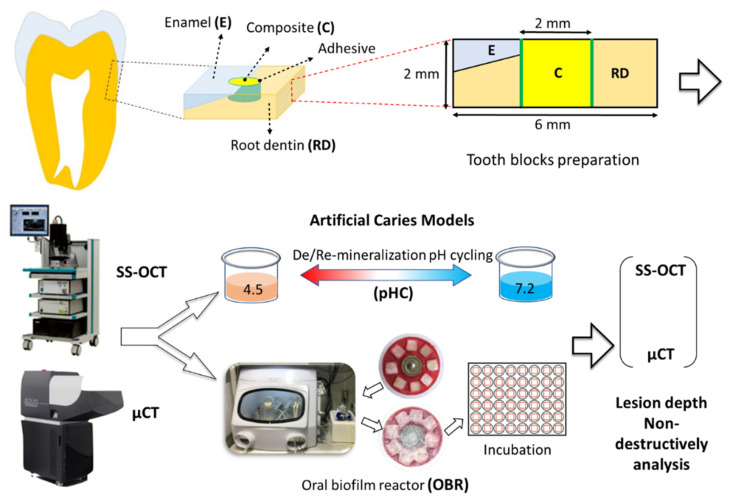
Schematic view of specimen preparation and study design for the anti-demineralization test. Thirty-two extracted human teeth were used in this study. Sixty-four cubical blocks (6 mm × 6 mm × 2 mm) were prepared from the enamel–root dentin junction. After adhesive (Single Bond Universal or FL Bond II) application and composite (Beautifil II or Estelite) restoration, half of the specimens were incubated for seven days after *S. mutans* biofilm formation in an oral biofilm reactor (OBR model). The other half of the specimens were immersed in de/remineralization pH cycling solutions for eight day cycles (pHC model). The specimens were subjected to SS-OCT and μCT observation at baseline and after pHC or OBR tests.

Specimen preparation and workflow of the study illustrated in Figure 1. Thirty-two human extracted molars with intact crowns and roots were collected with informed consent and approved by the Ethics Committee of National Taiwan University Hospital. The molars were cut in half to separate the buccal from the palatal or lingual surface using a low-speed diamond saw (Isomet 1000; Buehler, Lake Bluff, IL, USA). Sixty-four cubical tooth blocks (6 mm × 6 mm × 2 mm) were prepared from the enamel–root dentin junction with a low-speed diamond saw under water coolant. A cylindrical cavity (2 mm in diameter × 2 mm in height) was prepared in the cervical portion of each tooth block by using a high-speed hand piece. The specimens were ground to flatness with #800, #1000, #1500, and #2000 grit abrasive papers in sequence under running water. Afterward, the specimens were cleaned in an ultrasonic water bath for 1 min. Two composite resins, Beautifil II^®^ (BEF) (Shofu Inc., Kyoto, Japan) and Estelite^®^ (EST) (Tokuyama, Tokyo, Japan) with two adhesive systems, Single Bond Universal^®^(SBU) (3M Espe, St. Paul, MN, USA) and FL Bond II^®^(FL) were used in this study (Table 1). All specimens were randomly divided into two experimental groups (N = 32): the pH cycling (pHC) model and the oral biofilm reactor (OBR) model. Each group included thirty-two specimens which were then randomly subdivided into four subgroups (n = 8): BEF/SBU, EST/SBU, BEF/FL, and EST/FL. Adhesives and resin composites were applied into the cavities according to the manufacturer’s instructions. The light curing unit used in this study was 1000 mW/cm_2_ output (VALO Cordless LED curing light, Ultradent). After composite resin filling, the surfaces of all specimens were polished to #2000 grit abrasive paper.

**Figure 2:**
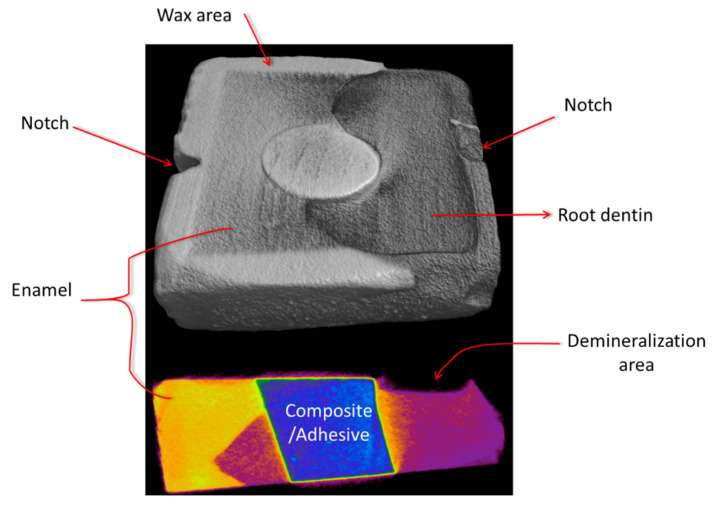
An example of 3D and 2D μCT images in the anti-demineralization test. Two notches on the two side of tooth block act as landmarks for SS-OCT.

All specimens were examined using SS-OCT (Santec OCT-2000, Santec Co., Komaki, Japan) to confirm that no overhangs existed. The samples were then rinsed with deionized water (Milli-Q water Systems, Millipore Corporation, Bedford, MA, USA) three times for two minutes each. Two notches were prepared on the margin of the tooth block as reference landmarks, and a thin layer of wax was applied approximately 1.0 mm from the restored cavity margin (Figure 2).

### 2.2. Establishment of the Artificial Caries Models

Two artificial caries models were set up: (1) pH cycling (pHC) model and (2) oral biofilm reactor (OBR) model, which can be compared to the others in this study. A brief description of these two models is as follows.

### 2.3. pH Cycling (pHC) Model

We prepared the demineralization (DM) and remineralization (RM) solution for the pHC model based on the instructions of a previous study [20]. The DM solution consisted of a mixture of 1.5 mM CaCl_2_, 0.9 mM KH_2_PO_4_, and 50 mM lactic acid. The pH value of the DM solution was adjusted to 4.5 by KOH. The RM solution consisted of a mixture of 1.5 mM CaCl_2_, 0.9 mM KH_2_PO_4_, 130 mM KCl, and 20 mM HEPES (buffer), and the pH value was adjusted to 7.2 by KOH. The tooth blocks were kept individually in the DM solution for three hours (10 mL per block), and then in the RM solution for 20 h (10 mL per block). Before switching the solution, each specimen was cleaned with ddH_2_O twice by a shaker (Vortex-Genie, G560, SI-0236 2). This process was repeated daily for eight days [21].

### 2.4. Oral Biofilm Reactor (OBR) Model

In order to mimic the oral environment, another artificial caries model, an OBR with artificial biofilms induced secondary caries model was set up [22,23,24]. In this study, biofilms were formed on the resin composite surfaces using *Streptococcus mutans* (*S. mutans*, MT8148). A laboratory strain of cariogenic bacteria, *S. mutans* MT8148 strain (Biosafety level 1) was used for this *S. mutans*-induced in vitro model. After 16 h of preculture, fresh *S. mutans* was cultured with 5 mL of brain heart-infusion broth (BHI; Becton, Dickinson and Company, Sparks, MD, USA) at 37 °C under anaerobic conditions using an O_2_-absorbing and CO_2_-generating AnaeroPouch (Mitsubishi Gas Chemical Co. Inc., Tokyo, Japan) for 16 h. Then, bacterial cells were washed with sterile and chilled phosphate-buffered saline (PBS) by centrifuging (3900 g) at 4 °C for 20 min [25]. A final suspension was prepared at an optical density of 490 nm (OD_490_) = 2.5 (approximately 2.50 × 10^8^ CFU/mL) using a spectrophotometer (Model 680 Microplate Reader; Bio-Rad, Hercules, CA, USA) and stored at 4 °C. For growth cultivation of *S. mutans* biofilm, a solution of heart infusion broth (Becton Dickinson) with sucrose (1.0% final concentration; HI-sucrose) was utilized.

The OBR included two chambers, where each chamber contained a warm water jacket with constant interior temperature and an environment favorable for the facultative anaerobic Gram-positive bacteria to grow biofilms. In order to regularly monitor the pH values beneath the biofilm layer, a pH electrode was used to measure the pH values and data were recorded every thirty minutes. Specimens were placed on a Teflon holder around a pH electrode and fixed with red utility wax (GC, Tokyo, Japan). The biofilms would then attach and accumulate over the experimental surface of the specimens.

In this experiment, biofilms were formed inside the two chambers that were encircled with a water jacket with a 37 °C inner temperature. Each chamber was sealed with a silicon plug fitted with five stainless steel tubes (21-gauge). The five stainless steel tubes were connected to five silicon tubes and regulated by a computer-operated controller (EYELA EPC-2000, Tokyo Rika, Tokyo, Japan). One tube was used to collect the suspension of *S. mutans*, two to collect sucrose-HI, and the other two to collect PBS. All of these liquids were pumped into the chambers at 6 mL⁄h per tube. The mixture of all these liquids formed water domes over the Teflon holder.

The specimens with biofilms were further incubated for another seven days to initiate demineralization over enamel and root dentin. All specimens were kept in separate wells at 37 °C and sucrose-HI was supplemented on alternate days (every 18 h). Upon addition of new medium, the pH was elevated, but would gradually decrease with the activity of the biofilms. After seven days of incubation, all of the specimens were washed by sodium hydroxide and Milli-Q water three times for two minutes each, in order to remove the biofilm on the specimen surfaces.

### 2.5. Swept-Source Optical Coherence Tomography (SS-OCT)

The SS-OCT system (IVS-2000, Santec, Komaki, Japan) was utilized to examine the specimens before and after the seven day incubation period. It operated based on a frequency domain OCT technique that measured the magnitude and time delay of reflected light in order to construct a depth profile. The wavelength ranged from 1260 nm to 1360 nm centered at 1310 nm with a 20 kHz sweep rate [24]. The axial resolution was 12.0 μm and the lateral resolution was 17.0 μm. To evaluate the artificial caries lesion over enamel and root dentin, ImageJ (version 1.48; National Institutes of Health, Bethesda, MD, USA) was used to analyze the raw data of SS-OCT. The depths of lesion could be obtained from the SS-OCT images along two marked points.

### 2.6. Micro Computed Tomography (ΜCT) Imaging Analysis

We examined the artificial caries lesions using μCT (SkyScan 1176, Bruker, Belgium). Measurements were taken before and after the experiments. Sixty-four samples were analyzed under μCT scanning with the setup of 65 kVp and 8.88 μm pixel. In order to evaluate the artificial caries lesion over enamel and root dentin, the obtained dataset was reconstructed with the CTAn 3D imaging system (SkyScan, Aartselaar, Belgium) and ImageJ (version 1.48; National Institutes of Health, Bethesda, MD, USA) to quantify and quality the artificial caries lesion. The region of interest (ROI) was confined to a cuboid area (3 mm × 3 mm × 0.5 mm).

### 2.7. Statistical Analysis

Statistical analyses were performed using the SPSS (Version 19.0.0). One-way ANOVA was the selected analytical method; the Scheffé test was used for post-hoc analysis. A *p*-value < 0.05 represented statistical differences. To determine the influencing factors, independent-samples *t*-test was used to analyze the influence of the composite resin materials or the adhesive materials on the specimens, respectively. Lesion depths obtained from SS-OCT and μCT were compared using Pearson’s correlation. The anti-demineralization test using pHC and OBR models were also compared by Pearson’s correlation.

## 3. Results

**Figure 3:**
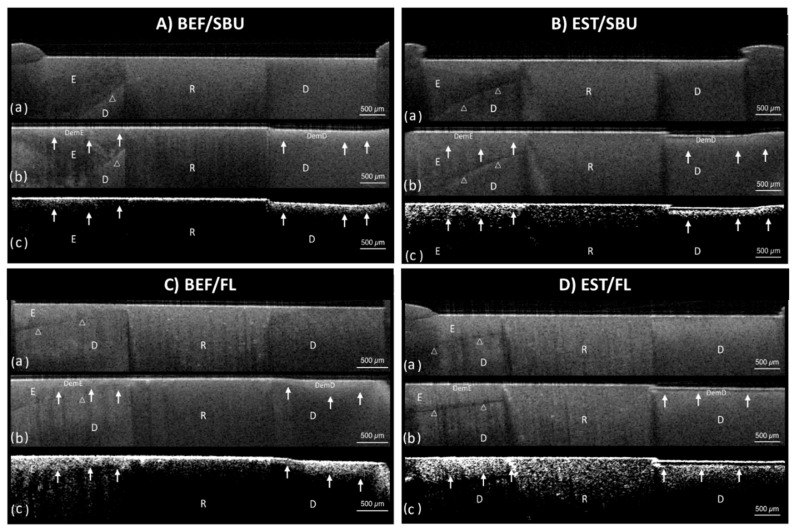
Representative cross-sectional image set of SS-OCT by de/re-mineralization cycling (pHC) model for four groups, (**A**) BEF/SBU, (**B**) EST/SBU, (**C**) BEF/FL, and (**D**) EST/FL group. (**a**) SS-OCT image before demineralization. A thin layer of wax was applied from the restored cavity margin. White-outlined arrowheads show the dentin–enamel junction. (**b**) SS-OCT image after demineralization. Solid white arrows show lesion boundaries under the demineralized enamel and demineralized root dentin. (**c**) SS-OCT image after the application of a noise reducing median filter. BEF—Beautifil II^®^ composite, EST—Estelite^®^ composite, SBU—Single Bond Universal^®^ adhesive, FL—FL Bond II^®^ adhesive, E—enamel, D—root dentin, R—resin.

**Figure 4:**
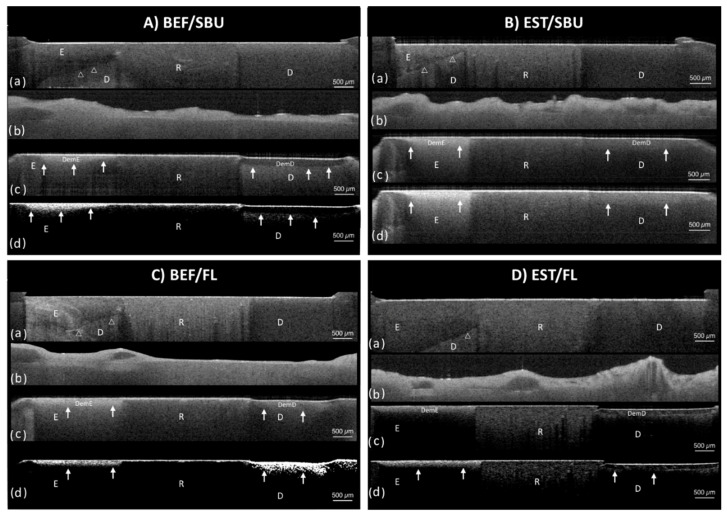
Representative cross-sectional image set of SS-OCT by oral biofilm reactor (OBR) model for four groups: (**A**) BEF/SBU, (**B**) EST/SBU, (**C**) BEF/FL, and (**D**) EST/FL group. (**a**) SS-OCT image before demineralization. A thin layer of wax was applied from the restored cavity margin. White-outlined arrowheads show the dentin–enamel junction. (**b**) SS-OCT image after 7 days incubation with *S. mutans* biofilm formation. (**c**) SS-OCT image after demineralization. Solid white arrows show lesion boundaries under the demineralized enamel and demineralized root dentin. (**d**) SS-OCT image after the application of a noise reducing median filter. BEF—Beautifil II^®^ composite, EST—Estelite^®^ composite, SBU—Single Bond Universal^®^ adhesive, FL—FL Bond II^®^ adhesive, E—enamel, D—root dentin, R—resin.

Representative cross-sectional SS-OCT images obtained from pHC and OBR models are shown in Figure 3 and Figure 4, respectively. For the artificial caries lesions, the backscattered signal increased and appeared as bright areas on the gray scale image while cavitation and complete loss of root dentin appeared as dark zones. The lesion boundary was observed between the brighter and darker regions.

### 3.1. Artificial Caries Pattern Assessment

The artificial caries lesions and the demineralization pattern of the lesions near the composite restoration over root dentin region were classified into four types based on the SS-OCT images through a single-blind randomized control trial which was defined as follows (Table 2):

(1) Type A: The lesion was generally shallow but became deeper away from the restoration. The bonding interface was intact and showed no attachment loss.
(2) Type B: Type B was similar to the Type A pattern except the lesion depth had a steeper drop near the restoration. The interface was still intact and showed no attachment loss.
(3) Type C: The depth of lesion was consistent whether near or away from the restoration. The interface presented significant attachment loss.
(4) Type D: The lesion depth was similar to Type C, however, the interface presented severe breakdown at the interface compared to Type C.

The percentage of each caries pattern group is summarized in Table 3. For the pHC model, the BEF/SBU group showed a predominantly Type B pattern, while the EST/SBU group exhibited a predominantly Type D pattern. Type A (37.5%) and Type B (62.5%) were observed in the BEF/FL group; while Type C (75%) and Type D (25%) were identified in the EST/FL group. For the OBR model, the BEF/SBU group presented three caries patterns: Type A (37.5%), Type B (37.5%), and Type C (25%). The EST/SBU group included Type C (25%) and Type D (75%), the BEF/FL group included Type A (75%) and Type B (25%), and the EST/FL group showed 62.5% of Type C and 37.5% of Type D.

### 3.2. Lesion Depths Assessment by SS-OCT and μCT

**Figure 5:**
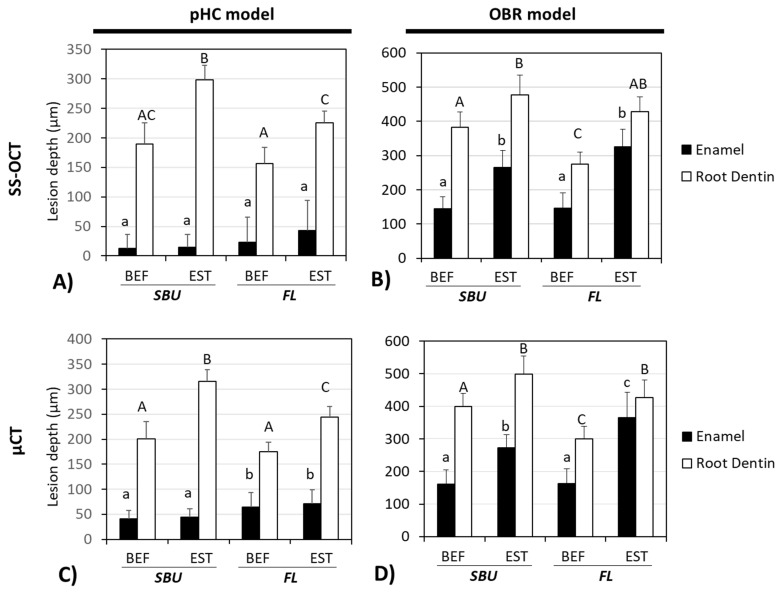
SS-OCT and μCT assessments of lesion depths of pHC model (**A**,**C**) and OBR model (**B**,**D**). Groups labeled with different letter superscripts showed significant differences (*p* < 0.05). BEF—Beautifil II^®^ composite, EST—Estelite^®^ composite, SBU—Single Bond Universial^®^ adhesive, FL—FL Bond II^®^ adhesive. pHC—pH cycling model, OBR—oral biofilm reactor model.

Figure 5A shows the pHC lesion depths measured from SS-OCT dataset. Lesions at the enamel region revealed no significant difference between the four groups (*p* > 0.05). Figure 5B shows the lesion depth from the OBR model from the SS-OCT dataset. For the enamel region, the Beautifil II composite containing groups (i.e., BEF/SBU = 144.9 ± 35.2 μm, BEF/FL = 145.7 ± 44.7 μm) showed significantly less lesion depth than the Estelite composite groups (*p* < 0.05). In the root dentin region, the BEF/FL group demonstrated the shallowest lesion depth (275.4 ± 34.6 μm) (*p* < 0.05). The lesion depth measured from μCT (Figure 5C,D) showed a similar tendency to SS-OCT (Figure 5A,B). Overall, the pHC model presented less lesion depth compared to the OBR lesion, especially in the enamel region.

### 3.3. Correlation Analysis of pHC and OBR Model, SS-OCT, and μCT

**Figure 6:**
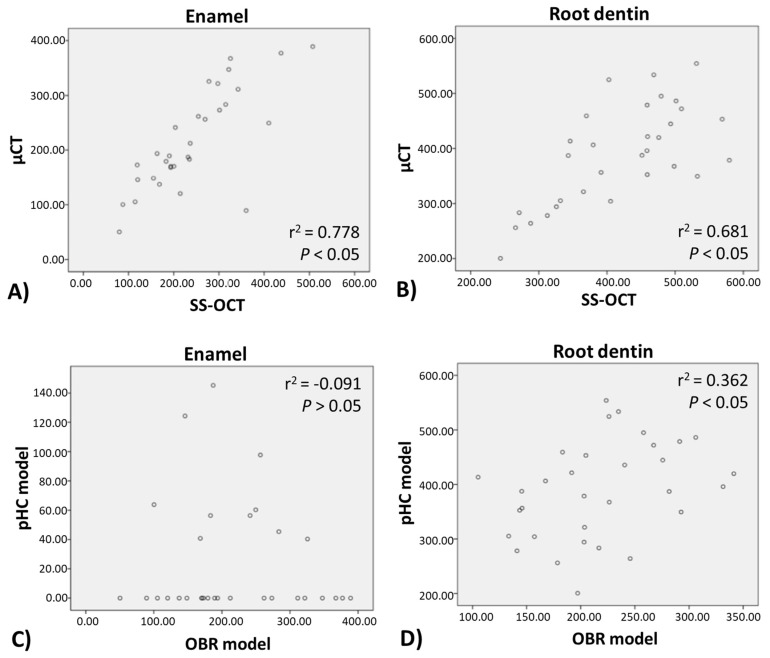
Pearson correlation coefficient analysis between the two evaluation methods (μCT and SS-OCT) and the two artificial caries models (pHC and OBR). The lesion depth measured from SS-OCT and μCT showed significant correlation (*p* < 0.05) for both (**A**) enamel and (**B**) root dentin. For artificial caries generated model, Pearson correlation coefficient showed no relationship between pHC and OBR model at (**C**) enamel region (*r*^2^ = −0.091, *p* > 0.05), but positive correlation at (**D**) the root dentin region (*p* < 0.05).

Correlation analysis (Figure 6) showed a significant relationship between SS-OCT and μCT values of the lesion depth over both the enamel region (*r*^2^ = 0.778, *p* < 0.05) and the root dentin region (*r*^2^ = 0.681, *p* < 0.05). It also demonstrated a significant relationship between pHC and OBR models over the root dentin region (*r*^2^ = 0.362, *p* < 0.05). However, lesion over the enamel region produced by the pHC and OBR models showed no significant correlation (*r*^2^ = −0.091, *p* > 0.05).

## 4. Discussion

The results of this study showed that tooth restoration combining both the SPRG-filler-containing composite and SPRG-filler-containing adhesive exhibited a superior anti-demineralization effect compared to the non-SPRG-filler-containing composite and adhesive, thus our hypothesis (1) was proven. Using composite or adhesive containing SPRG-filler alone can also resist demineralization, and significantly help to protect the integrity of the filling interface. The sustained fluoride release from pre-reacted glass particles (SPRG-fillers) has been proven to play a vital role in the prevention of secondary caries [26]. The sustained fluoride release from the denture when combined with a regular fluoride recharge regimen would produce a long-term effect of sustained fluoride release that may be beneficial for caries prevention [27]. Furthermore, the synergistic effect of the ions released by the SPRG-fillers also contributes to the antibacterial performance [9].

In this study, we employed two artificial caries model, the pHC and the OBR model, to evaluate the anti-demineralization effect of resin composites and adhesives. We found that the pHC model presented a much shallower lesion depth compared to the OBR model (about 1/3–1/8 of the OBR enamel lesion depth, e.g., 43 μm vs. 325 μm of the EST/FL group) and did not have a correlation with the OBR model at the enamel region (*r*^2^ = 0.091, *p* > 0.05, Figure 6C), while at the root dentin region, they still exhibited moderate correlation (*r*^2^ = 0.362, *p* < 0.05, Figure 6D). The root dentin lesion depth of the pHC model was about 3/5 of that produced by the OBR model (e.g., 156 μm vs. 275 μm of the BEF/FL group). Thus, we partially rejected hypothesis (2), as the pHC model did not display similar artificial caries pattern as the OBR model at the enamel region, but had moderate correlation at the root dentin region. In this study, the OBR model simulated the conditions of an oral environment by interacting with *S. mutans*, which is one of the main bacterial species associated with dental caries. *S*. *mutans* species secretes organic acids that can be trapped within the insoluble glucan matrix produced by bacteria, causing a locally prolonged drop in pH on tooth surface. *S. mutans* also displays strong adhesion to composite resin materials that do not possess anti-bacterial property [28,29]. *S. mutans* exhibits esterase activities that have the potential for biodegradation and can break down the adhesive–composite interface, thus forming micro-gaps [13] and generating secondary caries [30]. In the pHC model, the mechanism of pH alteration differs from the OBR model. Without the presence of bacteria, the pHC model does not produce a glucan matrix for adherence to the tooth or composite surface. Thus, the enamel surface without the trapped organic acid biofilm accumulation (glucan matrix) would be more resistant to acid attack and easier to remineralize. This is why the pHC model presents a much shallower lesion depth in the enamel than the OBR model, and the two models are incomparable in the enamel part. Yamamoto et al. reported that the SPRG-filling-containing material had the ability to inhibit demineralization [19]. While the SPRG-filler-containing composite combined with the use of the SPRG-filler-containing adhesive exhibited a superior demineralization resistance, the SPRG-filler-containing composite portion played a more significant role than the SPRG-filler-containing adhesive (Figure 5). The Beautifil II containing group always showed no attachment loss and maintained an intact joint of composite–adhesive (Table 3).

An intact bonding interface would less likely be destroyed by acid demineralization, further reducing the chance of recurrent caries. Single Bond Universal (SBU), one of the test adhesives in the study, has a 10-methacryloxydecyl phosphate (10-MDP) monomer, which contains a phosphate group that can readily form chemical bonds with the calcium in hydroxyapatite to achieve a higher bond strength and form a more intact and stable dentin-bonding joint [31]. The 10-MDP monomer containing adhesive systems also showed the capacity to form an acid–base resistant zone at the adhesive interface, which can counter acid–base challenges [32]. In this study, the dentin–bonding interface formed by SBU was not conducive to resisting acid erosion (Table 3, EST/SBU: 100% Type D of pHC model, 75% Type D of OBR model). However, when combined with a SPRG-filler-containing composite, the severity of attachment loss would lessen.

In this study, we analyzed an artificial caries lesion with SS-OCT and μCT. Overall, the lesion depth data obtained from SS-OCT were highly correlated to the data obtained from μCT (Figure 5 and Figure 6). Both μCT and SS-OCT have their own respective features and limitations as image evaluation tools. μCT is limited by the X-ray radiation range when evaluating an object’s cavitation depth, while SS-OCT is restricted by its depth of light penetration. Since SS-OCT relies on lasers to penetrate into the object to create clear images, it cannot be used for cavitation or fillings that are too deep. The samples in our study were 2 mm thick, and within the SS-OCT’s imaging working range of 2–3 mm [33]. In comparison to μCT, SS-OCT’s advantages include a shorter analysis time, ease of operation, applicability in a clinical setting, and is less costly. In addition, SS-OCT is an efficient tool for the detection and diagnosis of early caries lesions. The caries lesion could be efficiently evaluated utilizing SS-OCT image analysis, with regions of brightness signifying area of demineralization. In general, increased level of tooth structure demineralization corresponded to increased number of holes and mineral loss at lesion sites. A large number of microinterfaces between water and demineralized mineral crystals or demineralized collagen fibers in the pores leads to an increase in the backscattering (reflection) of light [34]. Therefore, a strong signal would be generated over the lesion sites, depicting areas of brightness in the corresponding SS-OCT image. Based on the given results shown in μCT and SS-OCT, the pHC model may not completely replace the OBR model since the OBR model’s bacterial system creates a more realistic simulation of the oral environment and can mimic the antibacterial and acidification effects against bacteria. However, the pHC model is better than the OBR system in terms of cost and convenience. The discrepancy between the two artificial caries models may be caused by the difference in the mechanism and time point of demineralization, which may require further investigation. The use of different concentrations and frequency of acid–base cycling, or prolonging the soaking time of the specimen may be able to overcome this limitation of the pHC model.

## 5. Conclusions

The use of the SPRG-filler-containing adhesive (i.e., FL Bond II) alone cannot effectively reduce the demineralization of enamel, but if the SPRG-filler-containing composite is used in combination, the cumulative synergy effect for anti-demineralization becomes significantly higher. The restorations using 10-MDP containing adhesive (i.e., Single Bond Universal) with the composite resin filling appeared to reduce the demineralization of enamel, but had no obvious effect when used on the root dentin. It is also worth noting that the composite resin containing SPRG-filler (such as Beautifil II) performed exceptionally in resisting acid attack and thus has good potential for recurrent caries prevention.

## Figures and Tables

**Table 1:** Materials used in this study.

Materials (Abbreviation)		Composition	Application Instructions
Adhesive	Single Bond Universal^®^ (SBU) 3M Espe, USA; Lot. 620316	10-MDP phosphate monomer, Vitrebond™ copolymer, HEMA, Bis-GMA, dimethacrylate resins, filler, silane, initiators, ethanol, water.	Apply the adhesive and rub it in for 20 s. Gently air dry for approximately 5 s. Light cure for 10 s
	FL Bond II^®^ (FL) Shofu, Kyoto, Japan; Lot. 101770 (primer) Lot. 101704 (bonding)	Primer: Water, ethanol, carboxylic acid monomer, phosphoric acid monomer and initiator. Adhesive: SPRG based on fluoroboro-aluminosilicate glass, UDMA, TEGDMA, 2-HEMA, initiator.	Apply primer, leave undisturbed for 10 s, air dry. Apply bonding agent, do not air dry. Polymerize for 5 s with LED.
Composite	Beautifil II^®^ (BEF) Shofu, Kyoto, Japan; Lot. 081695	S-PRG fillers 68.6% w/v 83.3% w/w Bis-GMA, TEGDMA	Dispense in layers up to 2 mm in thickness. Light cure for 20 s.
	Estelite^®^ (EST) Tokuyama, Tokyo, Japan; Lot. E054	Bis-GMA, TEGDMA Fillers: 82% wt, zirconia/silica particles	Dispense in layers up to 2 mm in thickness. Light cure for 20 s.

Abbreviations: Bis-GMA—bisphenol A diglycidylmethacrylate; MDP—10-methacryloyloxydecyl dihydrogen phosphate; HEMA—2-hydroxyethyl methacrylate; TEGDMA—triethylene glycol dimethacrylate; UDMA—urethane dimethacrylate.

**Table 2:** Artificial caries pattern: demineralization pattern of the lesions near the composite restoration over root dentin region by using SS-OCT.

Classification	Demineralization Pattern	Description	Attachment
Type A	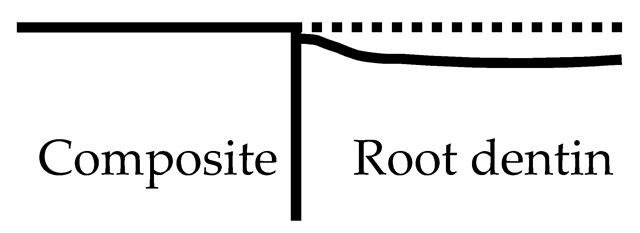	The lesion is shallower near the restorations and deeper away from the restoration.	No Attachment loss
Type B	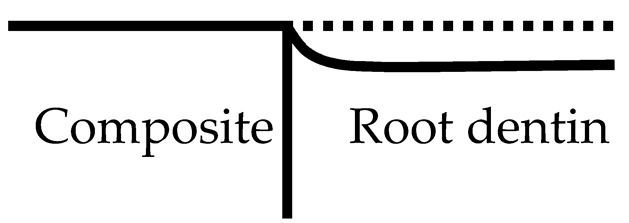	Type B is similar to Type A, but the lesion had a steeper drop near the restoration.	
Type C	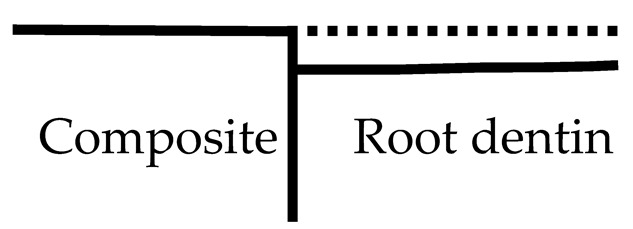	The lesion is of similar depth regardless of distance from the restoration.	Attachment loss
Type D	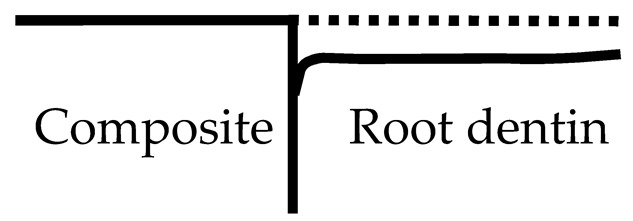	The lesion is inverted: Narrow and deep along the restoration.	

**Table 3:** Percentage of the artificial caries pattern.

Test Groups	Type A	Type B	Type C	Type D
**pHC Groups**				
BEF/SBU	–	100%	–	–
EST/SBU	–	–	–	100%
BEF/FL	37.5%	62.5%	–	–
EST/FL	–	–	75%	25%
**OBR Groups**				
BEF/SBU	37.5%	37.5%	25%	–
EST/SBU	–	–	25%	75%
BEF/FL	75%	25%	–	–
EST/FL	–	–	63.5%	37.5%

pHC—pH cycling, OBR—oral biofilm reactor, BEF—Beautifil II^®^ composite, EST—Estelite^®^ composite, SBU—Single Bond Universal^®^ adhesive, FL—FL Bond II^®^ adhesive.

## Data Availability

The data presented in this study are available on request from the corresponding author.
